# Strong Fermi-Level Pinning in GeS–Metal Nanocontacts

**DOI:** 10.1021/acs.jpcc.2c02827

**Published:** 2022-06-29

**Authors:** Yuxuan Sun, Zhen Jiao, Harold J. W. Zandvliet, Pantelis Bampoulis

**Affiliations:** Physics of Interfaces and Nanomaterials, MESA^+^ Institute for Nanotechnology, University of Twente, P.O. Box 217, 7500AE Enschede, The Netherlands

## Abstract

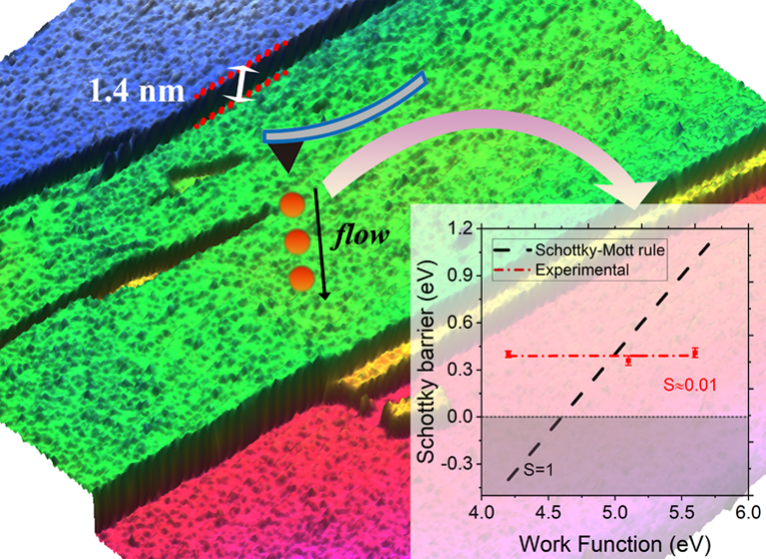

Germanium sulfide (GeS) is a layered monochalcogenide semiconductor with a band gap of about 1.6 eV. To verify the suitability of GeS for field-effect-based device applications, a detailed understanding of the electronic transport mechanisms of GeS–metal junctions is required. In this work, we have used conductive atomic force microscopy (c-AFM) to study charge carrier injection in metal–GeS nanocontacts. Using contact current–voltage spectroscopy, we identified three dominant charge carrier injection mechanisms: thermionic emission, direct tunneling, and Fowler–Nordheim tunneling. In the forward-bias regime, thermionic emission is the dominating current injection mechanism, whereas in the reverse-bias regime, the current injection mechanism is quantum mechanical tunneling. Using tips of different materials (platinum, n-type-doped silicon, and highly doped p-type diamond), we found that the Schottky barrier is almost independent of the work function of the metallic tip, which is indicative of a strong Fermi-level pinning. This strong Fermi-level pinning is caused by charged defects and impurities.

## Introduction

Efficient charge injection from metal contacts to low-dimensional materials, such as graphene and two-dimensional (2D) semiconductors, is of key importance for the successful implementation of these materials into electronic devices.^1−6^ Despite remarkable progress in the characterization and understanding of 2D materials, the fabrication of low-resistance contacts on 2D materials is still challenging. Ohmic contacts cannot be easily achieved by simply choosing the “right” metal. Non-ohmic contacts lead to inefficient devices with fluctuating performances. Fermi-level pinning (FLP) has been identified to be a major factor in this problem.^7^ FLP in contacts on low-dimensional materials has been linked to metal-induced gap states (due to orbital hybridization with the semiconductor), metal-induced defects, and intrinsic defects in the semiconductor.^8−11^

Among the two-dimensional materials, layered monochalcogenides are a very interesting class of materials. This is because they exhibit a rich variety of electronic properties depending on their composition and thickness.^12−15^ Layered monochalcogenides have band gaps comparable to that of silicon, anisotropic physical properties,^16−18^ strong spin–orbit couplings, and high charge carrier mobilities.^12,13,15^ In the past decade, several studies have scrutinized the possibility to use chalcogenides as the base material for field-effect-based electronic devices.^13,14^

Germanium sulfide (GeS) is a layered monochalcogenide material with an orthorhombic crystal structure with lattice constants of 0.365, 0.435, and 1.044 nm, respectively. The unit cell is centered rectangular.^18,19^ The interlayer spacing of GeS is given by the lattice constant in the direction normal to the two-dimensional layer of 1.044 nm plus the van der Waals gap of about 0.3–0.4 nm.^20^ The anisotropy in the crystal structure of GeS makes it an excellent candidate to study anisotropic physical properties.^21,22^ Moreover, GeS is expected to have high carrier mobilities, spin–orbit interactions, and thickness- and strain-dependent optoelectronic properties, such as band gap and excitonic states.^23−26^ Furthermore, the weak interlayer coupling in GeS, which allows for easy exfoliation of single- and few-layer flakes, and the stability at ambient conditions^18,27,28^ makes GeS a very appealing material for optoelectronic applications.^19,28−36^

However, realizing high-performance device applications based on GeS requires the fabrication of low-resistance ohmic contacts. In this work, we demonstrate that the fabrication of ohmic contacts on multilayer GeS cannot be easily achieved by just choosing a metal. Using the conductive tip of c-AFM as a metal electrode, we were able to investigate the injection of charge carriers in various metal–GeS nanocontacts. We found that the charge injection mechanism depends on the applied voltage bias and can be described by thermionic emission, direct tunneling, and Fowler–Nordheim tunneling. Current–voltage *I*(*V*) spectroscopy using a variety of tip materials with substantially different work functions, ranging from 4.2 to 5.6 eV, revealed that the Schottky barrier height at the interface is independent of the work function of the tip as a result of strong Fermi-level pinning by charged defects and impurities.

## Methods

In this study, we have used an Agilent 5100 atomic force microscope (AFM) in N_2_ environment (<0.1% relative humidity) to study the metal–GeS junction using various conductive tips. The GeS sample was purchased from HQ graphene (Groningen, The Netherlands), and the clean surfaces were prepared by mechanical exfoliation using the scotch tape method prior to scanning. The GeS sample was subsequently mounted on a Mo sample holder. Hereafter, the transport properties of the nanoscale junction were investigated using the conductive mode of the AFM. In the conductive atomic force spectroscopy mode, the conductive AFM tip (grounded) is brought into contact with the semiconductor surface, and subsequently, the sample bias voltage, *V*, is ramped and the current, *I*, is recorded. The characteristic shape of the *I*(*V*) traces provides direct information on the current injection mechanism. The experiments were done with three different metallic AFM tips: a highly *p*-doped diamond tip (AD-E-2.8-SS; Adama Innovations Ltd.) with a work function of 5.1 ± 0.1 eV,^37^ a strongly n-type silicon tip (Hi’Res-C14/Cr-Au, MikroMasch) with a work function of 4.2 ± 0.1 eV,^9,10,38^ and a Pt tip (12Pt400B, Rocky Mountain Nanotechnology LLC) with a work function of 5.6 ± 0.1 eV.^11,39^ The tip work functions have been also confirmed using the Kelvin probe force microscopy mode using a Au calibration sample. The nominal spring constants of the p-type diamond tip, n-type Si tip, and Pt tip are 0.5, 2, and 0.3 N/m, respectively. The radii of curvature of the p-type diamond tip, n-type Si tip, and Pt tip are 10 ± 5, 1.5 ± 0.5, and 15 ± 5 nm, respectively.

## Results and Discussion

**Figure 1:**
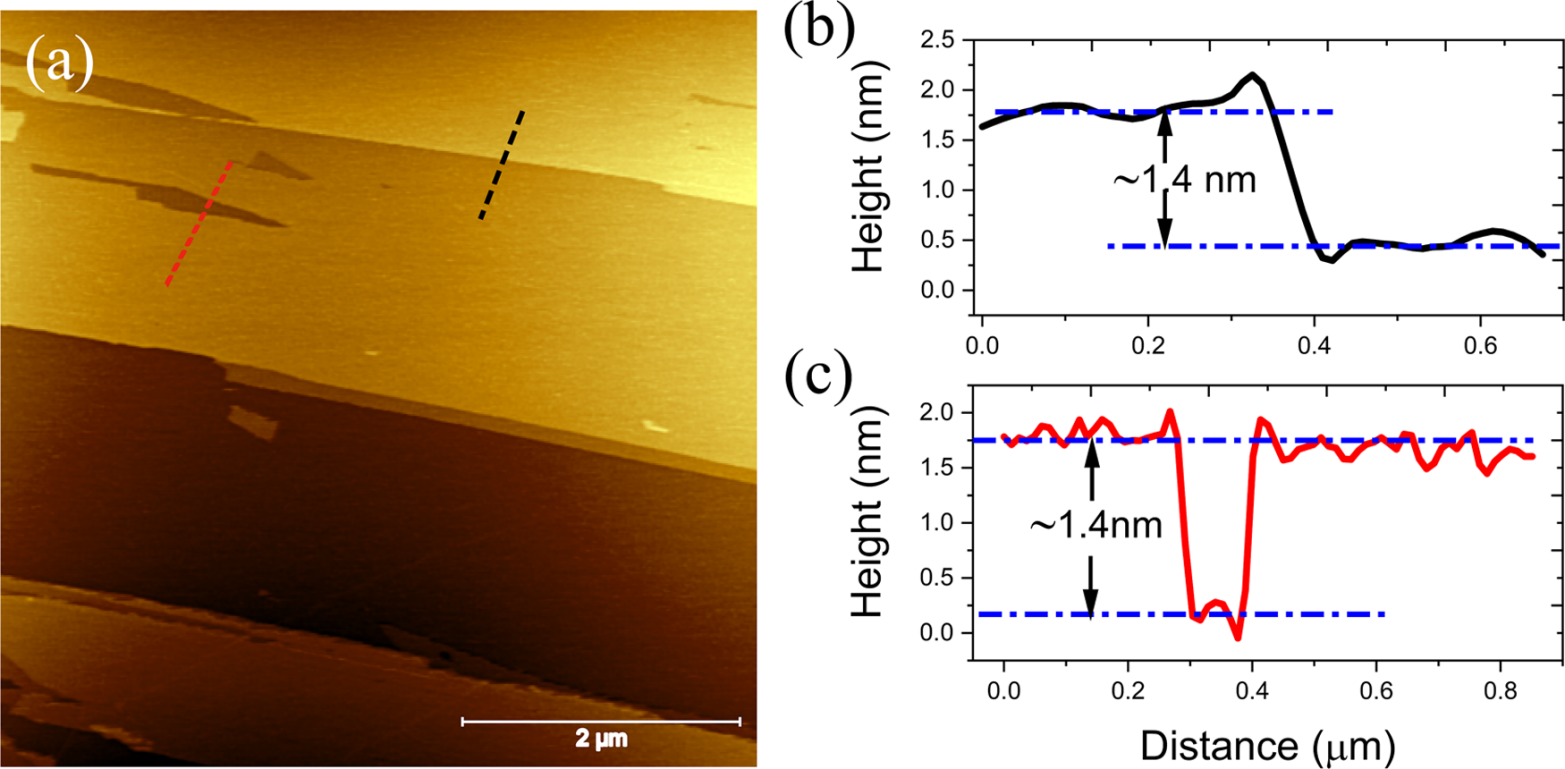
(a) AFM topography image of GeS. (b) Line scan, corresponding to the dashed black curve in (a), across a single layer step of GeS. (c) Line scan, corresponding to the dashed red curve in (a), across a vacancy island.

The AFM topography of a freshly exfoliated GeS sample is shown in Figure 1a. The exfoliated GeS surface contains a large number of step edges, tiny flakes, and vacancies. The height of these flakes and the depth of these vacancies correspond to a single GeS layer (see Figure 1b,c). The presence of these small flakes and vacancies or depressions suggests that the intralayer interaction in GeS is not as strong as some other 2D materials, like graphene and molybdenum disulfide, where flakes and vacancies are usually fully absent.

**Figure 2:**
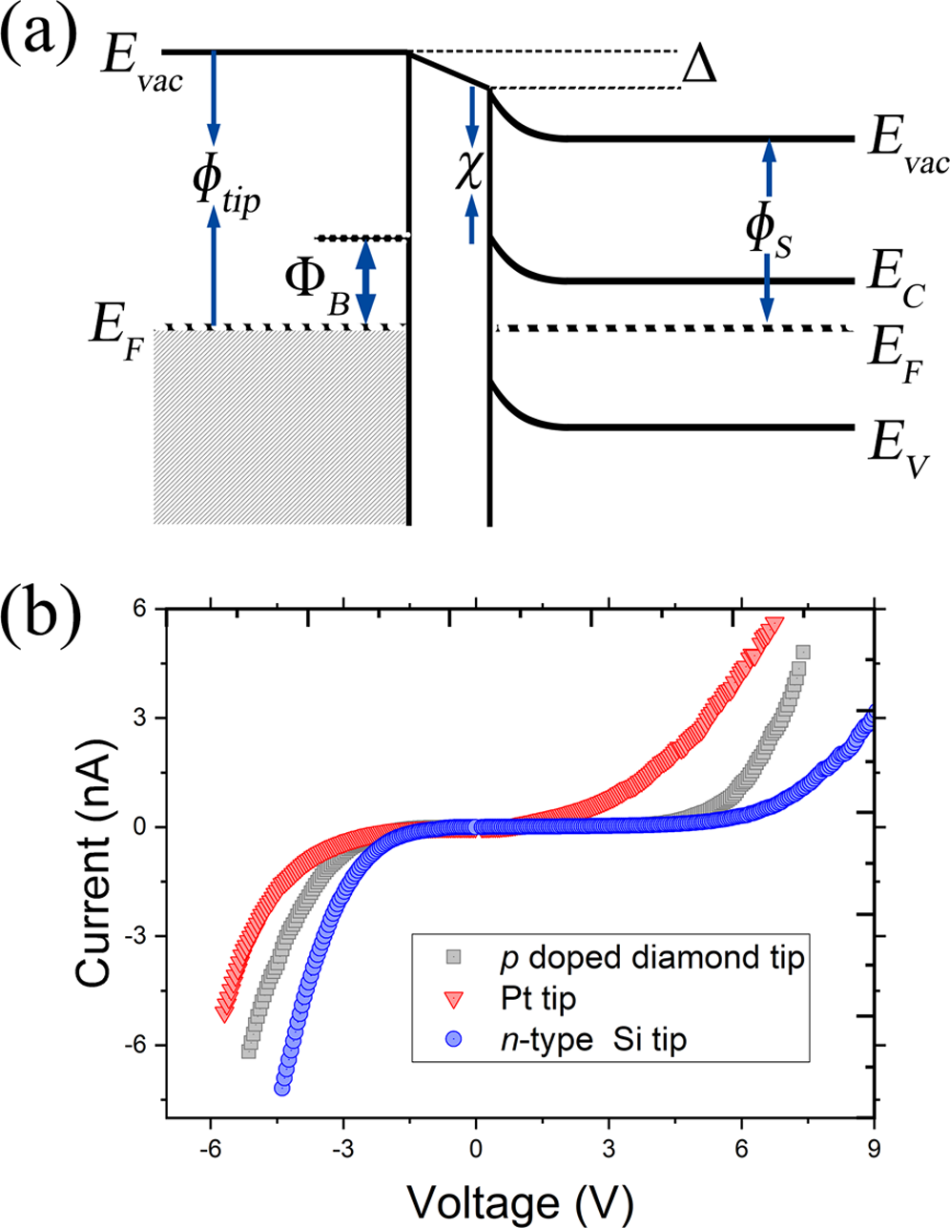
(a) Band structure of the metal/GeS junction at equilibrium. (b) Contact *I*(*V*) curves recorded on GeS with Pt, n-Si, and p-diamond tips.

A metal nanocontact on GeS can be established by placing a metallic tip in direct contact with the GeS substrate. When the metal tip and GeS substrate are brought into contact, electrons will flow from the material with the lowest work function to the material with the highest work function until the Fermi levels of both materials become equal. The alignment of the Fermi levels gives rise to a charge redistribution at the metal–semiconductor interface and the formation of the Schottky barrier, as shown in Figure 2a. The height of this Schottky barrier for ideal n-type contacts is given by the Schottky–Mott rule, Φ_B_ = ϕ_tip_ – χ, where ϕ_tip_ is the work function of the metallic tip and χ is the electron affinity of GeS. However, the presence of interface states (Figure 2a), which are induced by defects (defect-induced gap states) or metal–semiconductor orbital hybridization (metal-induced gap states), can lead to deviations from the Schottky–Mott rule. It is also very likely that there exists a very thin tunnel barrier between the metallic tip and the GeS layer (see Figure 2a). The p-type diamond, n-type Si and Pt tips have work functions that are close to the expected bulk values of 5.1 ± 0.1,^37^ 4.2 ± 0.1,^9,10,38^ and 5.6 ± 0.1 eV,^11,39^ respectively. The bulk GeS electron affinity is 3.8 eV.

Figure 2b shows three contact *I*(*V*) curves obtained, respectively, with p-type diamond, n-type Si, and Pt tips in contact with the GeS surface. Each of these *I*(*V*) curves are the result of averaging about 100 single-point curves obtained at various locations on the surface. Therefore, they represent the average behavior of the GeS surface. The curves have an asymmetric appearance. The current flows nonlinearly in both voltage directions, where the current increases sharper for negative sample biases than for positive sample biases, suggesting that the negative bias is the forward-bias regime for all three junctions.

Charge transport through the Schottky barrier can occur through thermionic emission, direct quantum mechanical tunneling, or Fowler–Nordheim tunneling.^40−44^ In thermionic emission (TE), the thermal energy of the electrons allows some of the electrons to overcome the Schottky barrier. For TE, the probability to overcome the Schottky barrier is given by the Boltzmann distribution, i.e., e^–Φ_B_/*kT*^. The current as a function of the applied sample bias across the metal–semiconductor junction is given by^9,43^
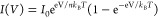
1where *T* is the temperature, *k*_B_ is the Boltzmann constant, *n* is the ideality factor, and *I*_0_ = AA*e^–Φ_B_/*kT*^ is the saturation current. *A* is the contact area, and A* is the Richardson constant given by , where m* is the effective mass (here m* = 0.5 m) and *h* is Planck’s constant. When the Schottky barrier is thin enough or a van der Waals gap exists between the semiconductor and the metal contact, quantum tunneling can become important too.^43^ The direct tunneling (DT) current as a function of the applied sample bias across a barrier with width *d* is given by^41,43^
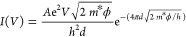
2where ϕ is the height of the tunnel barrier.

**Figure 3:**
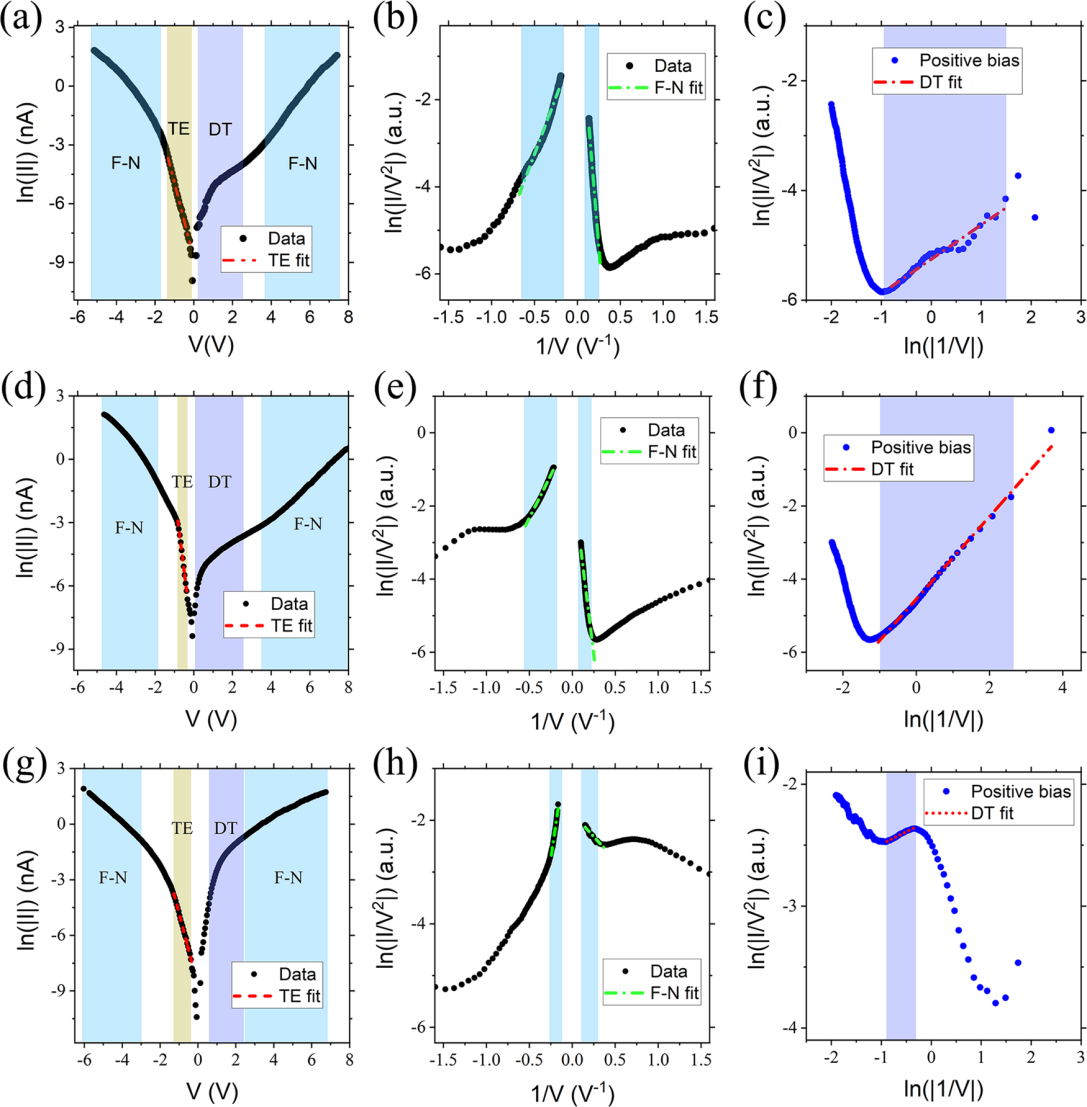
*I*(*V*) characteristics recorded with a p-type-doped diamond (top), n-type Si (middle), and Pt (bottom) tips plotted in three different ways. Left: ln(|*I*|) versus *V* plot, middle: ln(*I*/*V*^2^) versus 1/*V* plot (Fowler–Nordheim plot), and right: ln(*I*/*V*^2^) versus ln(1/|*V*|).

Fowler–Nordheim (FN) tunneling occurs at higher bias voltages. In FN tunneling, the electrons tunnel through a triangular-shaped barrier, which gets thinner and steeper with increasing bias. In the case of FN tunneling, the current as a function of the sample bias is given by^41,43^
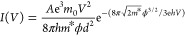
3A careful investigation of the characteristic shape of the *I*(*V*) traces, shown in Figure 2, can provide qualitative information on which of these injection mechanisms is dominant. By plotting ln(|*I*|) versus *V*, one finds, for bias voltages |eV| ≫ 3*kT*, a straight line, which is the hallmark for TE.^40^ This is the case for all three tip-GeS contacts at small biases and in the forward-bias regimes, as can be seen in Figure 3a–d,g. The slope of this line is equal to e/*nkT* and allows one to extract the ideality factor *n* using
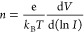
4The ideality factor refers to the deviation of the current transport from ideal thermionic emission. *A* value of 1 indicates excellent agreement. We find ideality factors in the range of 1 to about 5. Therefore, TE fits the data quite well. The height of the Schottky barrier can be extracted from the intercept, provided that the electrical contact area is known.
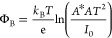
5The contact area between the tip and substrate is estimated using the Hertz model.^45−49^ The contact area between the p-type diamond tip (radius of curvature 10 ± 5 nm) and GeS is estimated to be 4 ± 1 nm^2^ at a load of ∼5 nN. The contact area between the n-type Si tip (radius of curvature 1.5 ± 0.5 nm) is estimated to be 10 ± 2 nm^2^ at a load of ∼200 nN. The contact area between the Pt tip (radius of curvature 15 ± 5 nm) is estimated to be 25 ± 5 nm^2^ at a load of ∼50 nN. A careful selection of the tip contact force was critical for establishing a stable contact area. The extracted barrier heights for the forward-bias regimes are 0.36 ± 0.03, 0.40 ± 0.03, and 0.41 ± 0.03 eV for the p-diamond, n-type Si, and Pt tip contacts, respectively. Fitting with TE was done in the range in the low-voltage forward-bias regimes. This way contributions from the substrate resistance can be avoided.^50^ We note here that instead of modeling the system as a metal–semiconductor metal contact,^51^ the second electrode (large silver paint contact) has been neglected. This is done because the large asymmetry in contact sizes (30 000 μm^2^ vs 10 nm^2^) makes the current blocked by the big contact negligible in the forward-bias regime.^52,53^

For much larger voltage biases (>2 V), the ln*(I)* versus *V* plots deviate from TE. In this voltage regime, FN tunneling is expected to dominate the charge transport. For this reason, we have plotted the *I*(*V*) curves as ln(|*I*|/*V*^2^) versus 1/*V* (see Figure 3b–e–h). This type of plot is often referred to as the Fowler–Nordheim plot. Using eq 6, we have extracted the barrier parameter *d*ϕ^3/2^ by fitting the curves in the linear high-voltage bias regime (indicated in Figure 3b–e–h). The extracted barrier parameters are summarized in Table 1.
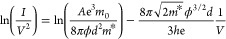
6TE fits the data well at small forward voltages (albeit with an ideality factor that exceeds 1), and FN describes the data at large voltage biases (range) for both forward and reverse biases. Charge transport at small reverse biases can be on the other hand ascribed to DT. In the case of DT, it is most convenient to plot ln(|*I*|/*V*^2^) versus ln(1/*V*), as shown in Figure 3c–f–i. From the slope of the straight line and the intercept, the barrier parameter *d*√ϕ can be extracted using eq 7
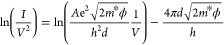
7Indeed, DT fits the data in small reverse biases. The DT barrier parameters are summarized in Table 1.

**Table 1:** Experimental Barrier Data of Metal–GeS Nanocontacts Obtained for Different AFM Tips (p-Type-Doped Diamond, n-Type-Doped Si and Pt)^a^

barrier	TE Φ_B_ [eV]	DT *d*√ϕ [eV^1/2^ nm]	FN ϕ^3/2^*d* [eV^3/2^ nm]	
tip	*V*_S_ < 0	*V*_S_ > 0	*V*_S_ < 0	*V*_S_ > 0
n-type-doped Si ϕ = 4.2 eV	0.40 ± 0.03	0.6 ± 0.1	1.0 ± 0.1	4.0 ± 0.1
p-type-doped diamond ϕ = 5.1 eV	0.36 ± 0.03	0.7 ± 0.1	1.1 ± 0.1	5.0 ± 0.1
Pt ϕ = 5.6 eV	0.41 ± 0.03	0.3 ± 0.1	1.8 ± 0.1	0.4 ± 0.1

a TE, DT, and FN stand for thermionic emission, direct tunneling, and Fowler–Nordheim tunneling, respectively. *V*_S_ is the sample bias, *d* is the width of the barrier, ϕ is the tunnel barrier, and Φ_B_ is the Schottky barrier.

**Figure 4:**
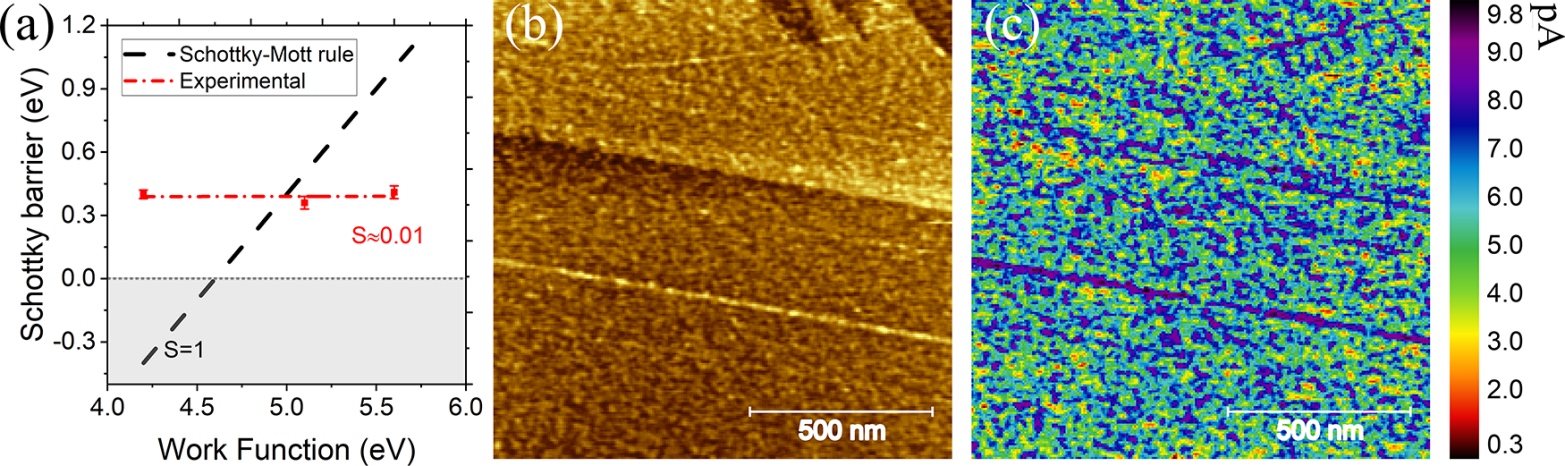
(a) Schottky barrier versus work function of the tip. The dashed black line is the Schottky–Mott relation ϕ_B_ = ϕ – χ (*S* = 1). The red dashed line shows the experimentally determined Schottky barrier. *S* = 0.01 implies a strong pinning of the Fermi level of GeS. (b) Small-scale AFM image and (c) current map of the same region as shown in (b).

To sum up, TE dominates at small forward biases (<1 V), FN tunneling at large biases (> 2 V), and DT at small reverse biases. The Schottky barrier heights range from 0.36 to 0.41 eV and do not depend on the work function of the metallic AFM tip. As shown in Figure 4a, in which the Schottky barrier heights are plotted as a function of the work function of the metal tip, the experimental results strongly deviate from what is expected from the ideal Schottky–Mott rule (black dashed line). The discrepancy can be understood by the fact that the Schottky–Mott rule does not take into account the presence of interface states, which could pin the Fermi level. Deviations from the ideal Schottky–Mott rule are common for both bulk and 2D semiconductors due to Fermi-level pinning at interface states induced either by defects or metal–semiconductor orbital hybridization. Considering FLP, the Schottky barrier height is given by
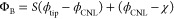
8where *S* is the pinning parameter  and ϕ_CNL_ is the charge neutrality level with respect to the vacuum level given by ϕ_CNL_ = (χ + *b*)/(1 – *S*), where *b* is the intercept of the barrier height versus work function plot. For *S* = 0, the Schottky barrier is independent of the tip work function and the Fermi level is pinned by the interface states at ϕ_CNL_. For *S* = 1, the Schottky–Mott rule is recovered. The pinning factor can be obtained from the slope of the barrier height versus work function plot. We find that the pinning factor for metal–GeS nanocontacts amounts to 0.01, indicative of very strong Fermi-level pinning, substantially stronger than in transition-metal dichalcogenides.^9,54−56^ The CNL was extracted to be located at 4.16 eV.

GeS is a two-dimensional van der Waals material and thus it is not expected to have any intrinsic surface states, i.e., dangling bonds. However, as can be seen in Figure 4b, small-scale AFM images of GeS are quite rough, with a mean-square roughness of about 0.5 nm. Moreover, current images such as the one in Figure 4c are electronically inhomogeneous, suggesting a high number of defects. In a similar way, defects were found to induce stronger FLP compared to pristine regions on TMDCs.^9,11,54,57^ Comparing with similar measurements on TMDCs, GeS appears to be way more defected, indicating a lower stability of the material to ambient conditions. Based on these observations, we arrive at the conclusion that the high number of defects on the exfoliated GeS is responsible for the strong Fermi-level pinning and the weak dependence of the Schottky barrier on the work function.

## Conclusions

We have studied the electronic transport mechanisms in metal–GeS nanocontacts. Depending on the applied potential difference between the metallic AFM tip and GeS, various current injection mechanisms are active. At small forward biases, thermionic emission is active. Direct tunneling takes over at reverse small biases and at applied potential differences exceeding about 2 V Fowler–Nordheim tunneling becomes the dominant current injection mechanism. We have found that the Schottky barrier of GeS varies from 0.36 ± 0.03 to 0.41 ± 0.03 eV for the different AFM tips. The Schottky barrier is almost independent of the work function of the metal, which is indicative of a strong Fermi-level pinning. This Fermi-level pinning is caused by charged defects and impurities in GeS, which result in the formation of interface states. The strong Fermi-level pinning on GeS nanocontacts suggests that alternative strategies^58,59^ must be used to obtain low-resistance ohmic contacts for efficient charge injection in GeS-based devices.
